# Lip Lift Video Roundtable

**DOI:** 10.1093/asjof/ojad059

**Published:** 2023-06-28

**Authors:** Foad Nahai, T Gerald O’Daniel, Francisco G Bravo, Chia Chi Kao

The authors are pleased to present an expert video roundtable discussion on lip lift (Video). The discussants include a panel of leading experts in aesthetic surgery, and the video roundtable was recorded live at The Aesthetic Meeting 2023 in Miami, FL, USA. The panelists discuss the factors that have contributed to the lip lift's recent popularity, the considerations surgeons must take when performing the procedure, and share their preferred techniques to achieve the most complimentary lip for each individual patient. Though lip lifts have been performed for decades, many patients have been turning to filler in place of a lip lift. However, while fillers do create the desired plumper lip, they can also make lips look puffy and heavy, which lately has motivated patients to seek out an alternative. But above all, the panelists agree that the lip lift has social media to thank for its recent resurgence, as patients emulate the full lips of the celebrities and influencers they see online.

Lip lifting has proven extremely beneficial to plastic surgeons because it targets an area that has historically been overlooked in favor of face and neck rejuvenation. The increase in rhinoplasties especially has drawn attention to upper lip length; indeed, a common effect of tip rotation is increased length of the upper lip, a phenomenon brought to the forefront by Perkins et al in their 2017 *Aesthetic Surgery Journal* article.^1^ A lip lift completes not only the rejuvenation, but also the beautification, of the face as a whole.

At the same time, it is important to understand that the lip lift is a long, complicated procedure, and careful consideration must be taken to determining the ideal technique for each patient. Our panelists agree that a lip lift requires a customized, tailored approach, and in fact, a lip lift may not be suited to everyone who desires one. The aesthetic benefit must outweigh the incision, and it is imperative that surgeons take all factors into account before committing to the procedure.

## Supplementary Material

ojad059_Supplementary_Data
